# The N-terminal Region of Nisin Is Important for the BceAB-Type ABC Transporter NsrFP from *Streptococcus agalactiae* COH1

**DOI:** 10.3389/fmicb.2017.01643

**Published:** 2017-08-29

**Authors:** Jens Reiners, Marcel Lagedroste, Katja Ehlen, Selina Leusch, Julia Zaschke-Kriesche, Sander H. J. Smits

**Affiliations:** Institute of Biochemistry, Heinrich Heine University Düsseldorf Düsseldorf, Germany

**Keywords:** ABC transporter, lanthionine ring, lantibiotic, nisin, resistance

## Abstract

Lantibiotics are (methyl)-lanthionine-containing antimicrobial peptides produced by several Gram-positive bacteria. Some human pathogenic bacteria express specific resistance proteins that counteract this antimicrobial activity of lantibiotics. In *Streptococcus agalactiae* COH1 resistance against the well-known lantibiotic nisin is conferred by, the nisin resistance protein (NSR), a two-component system (NsrRK) and a BceAB-type ATP-binding cassette (ABC) transporter (NsrFP). The present study focuses on elucidating the function of NsrFP via its heterologous expression in *Lactococcus lactis*. NsrFP is able to confer a 16-fold resistance against wild type nisin as determined by growth inhibition experiments and functions as a lantibiotic exporter. Several C-terminal nisin mutants indicated that NsrFP recognizes the N-terminal region of nisin. The N-terminus harbors three (methyl)-lanthionine rings, which are conserved in other lantibiotics.

## Introduction

Lantibiotics are ribosomally synthesized antimicrobial peptides of approximately 19–38 amino acids, which are mainly produced by Gram-positive bacteria (Klaenhammer, 1993). They are characterized by extensive post-translational modifications, which result in the presence of dehydrated amino acids, lanthionine and methyl-lanthionine rings (Chatterjee et al., 2005). Lantibiotics are considered to be promising candidates as antibiotic alternatives due to their capability to inhibit various multidrug-resistant pathogenic bacteria such as Staphylococci, Enterococci, Streptococci and Clostridia species (Dischinger et al., 2014). Several lantibiotics are also effective against Gram-negative bacteria like species of the *Neisseria* and *Helicobacter* genus (Mota-Meira et al., 2000). The pharmaceutical potential of lantibiotics has been extensively studied and some are already in the preclinical and clinical phases of development (Yang et al., 2014). Lantibiotics exhibit different modes of action including binding to the cell wall, which results in growth inhibition, as well as subsequent pore formation leading to immediate cell death (Brötz et al., 1998a; Hasper et al., 2004, 2006; Islam et al., 2012).

Some bacteria, however, are inherently resistant against lantibiotics due to the expression of various protein systems that can detect and subsequently respond to the presence of lantibiotics in the extracellular medium (reviewed in Draper et al., 2015). These broad range resistance systems can either be unspecific such as changes in bacterial cell wall and membrane (Nawrocki et al., 2014; de Freire Bastos et al., 2015; Draper et al., 2015) or more specific by proteolytic degradation of the lantibiotic itself (Sun et al., 2009).

**FIGURE 1:**
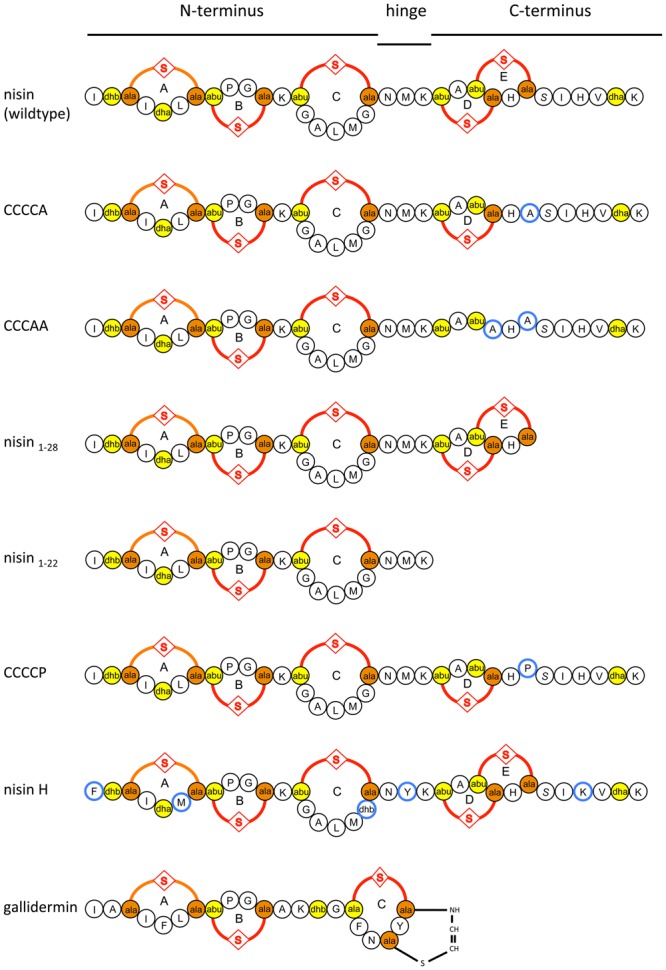
Schematic overview of nisin, the variants of nisin, nisin H, and gallidermin used in this study. Introduced mutations in CCCCA, CCCAA, CCCCP, and the natural variants in nisin H are highlighted in blue. The (methyl)-lanthionine rings (labeled with A, B, C, D, and E) are formed by a dehydrated amino acid residue and a cysteine residue side chain (orange and yellow).

In the present study, we focused on the lantibiotic nisin, which is produced by some *Lactococcus lactis* and *Streptococcus uberis* strains (Klaenhammer, 1993; Chatterjee et al., 2005). Nisin has a broad antimicrobial spectrum against a wide range of Gram-positive bacteria and exhibits several different modes of action (Ruhr and Sahl, 1985; Brötz et al., 1998b; Hsu et al., 2004; Hasper et al., 2006). One dominant activity is the binding to lipid II, a precursor molecule of peptidoglycan, thereby inhibiting cell wall synthesis (Wiedemann et al., 2001). Secondly, nisin is able to insert into the membrane to form pores (Hasper et al., 2004), which leads to the efflux of ions, nutrients, and subsequently to cell death. This last activity is a very rapid process and occurs almost instantly. Nisin can be structurally dissected in the N-terminus (containing the (methyl)-lanthionine rings A–C), a hinge region with the amino acids NMK and the C-terminus containing rings D and E (Van de Ven et al., 1991) (**Figure 1**). These rings are crucial for the nM activity and deletion of for example only ring E reduces the activity about eightfold (Alkhatib et al., 2014b).

Within the human pathogen *Streptococcus agalactiae* COH1 the expression of a proteogenous resistance system comprising of NSR (nisin resistance protein; a serine protease), an ATP-binding cassette (ABC) transporter (NsrFP) and a two-component system (TCS) (NsrRK) confers resistance against nisin (Khosa et al., 2013). Recently, this NSR operon has been characterized biochemically and structurally. *In vitro* studies showed that NSR expressed in *L. lactis* confers 20-fold resistance against nisin. This is mediated by cleaving off the last six amino acids from nisin, thereby lowering its activity (Sun et al., 2009; Khosa et al., 2013, 2016a). Another component of this nisin resistance operon is the BceAB-type ABC transporter NsrFP. BceAB-type ABC transporters, are putatively involved in antimicrobial peptide (like lantibiotics) removal from the lipid membrane (Gebhard and Mascher, 2011). They have been named after the BceAB transporter system from *Bacillus subtilis* conferring resistance against the antimicrobial peptide bacitracin (Ohki et al., 2003; Rietkötter et al., 2008). Interestingly, within the genomes the lantibiotic BceAB-type ABC transporter are encoded in close proximity to a TCS (Khosa et al., 2013) which senses the presence of the lantibiotic and subsequently up-regulates the expression of the ABC transporter (Dintner et al., 2011). The BceAB from *B. subtilis* has been shown to form a multicomponent complex with its designated TCS BceRS upon binding of bacitracin (Dintner et al., 2014). This highlights that the BceAB transporter from *B. subtilis* is directly involved in bacitracin sensing and consequently triggering the up-regulation of its own gene by the TCS BceRS.

**FIGURE 2:**
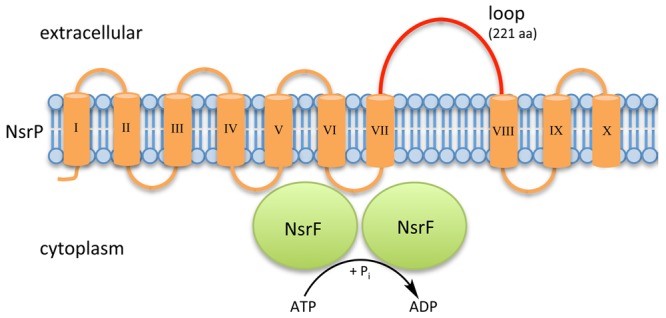
Schematic representation of NsrFP from *S. agalactiae* COH1. NsrFP is an ABC transporter consisting of NsrF (highlighted in green), which hydrolyses ATP and NsrP (highlighted in orange). NsrP is a membrane protein consisting of 10 predicted transmembrane helices with an extracellular domain (depicted in red), which is a characteristic for the BceAB-type of lantibiotic resistance transporters.

Within NsrFP from *S. agalactiae* COH1, the transmembrane domain NsrP contains 10 predicted transmembrane helices and harbors a 212 amino acid large extracellular domain (ECD) in between helices VII and VIII (Khosa et al., 2013; **Figure 2**). NsrF is the nucleotide-binding domain delivering the energy for the transport by ATP hydrolysis.

In this study, we determined the function of NsrFP from *S. agalactiae* COH1 in conferring nisin resistance. We expressed only the NsrFP transporter without the corresponding NsrR/NsrK TCS and observe that NsrFP can confer resistance up to 80 nM nisin. In comparison to this, a strain lacking this transporter can only survive a nisin concentration of 5 nM. Above this concentration the cells are suffering from pore formation mediated by nisin. Furthermore, we could show that NsrFP works as a lantibiotic exporter by a peptide release assay. Additionally several mutants of nisin were used to investigate the substrate specificity, which highlights that NsrFP recognizes the N-terminal region of nisin. This was confirmed by the observed resistance against nisin H (O’Connor et al., 2015) and gallidermin (Kellner et al., 1988), which both contain a similar N-terminus but differ in the C-terminal part of the peptide.

## Materials and Methods

### Cloning of *nsrfp*

The *nsrfp* gene from *S. agalactiae* COH1 was amplified from the chromosomal DNA using two primers (NsrFP_for 5′-CATCACCACCACCACTTATTAGAAATCAATCACTTAG-3′ and NsrFP_rev 5′-GTGGTGGTGGTGGTGCATATAATTCTCCTTTATTTATTATAC-3) and ligated into *pIL-SV* (*E. coli*–*L. lactis* shuttle vector) (Alkhatib et al., 2014b). The point mutation NsrF_H202A_ was introduced by a standard mutagenesis protocol using the following primers: forward: 5′-GATGGTAACCGCTTCAGCAAATGCTG-3′; reverse: 5′-CAGCATTTGCTGAAGCGGTTACCATC-3′. The resulting plasmid was verified by sequencing and transformed into the *L. lactis* strain NZ9000 for expression (Holo and Nes, 1989) and the corresponding strains were termed NZ9000NsrFP and NZ9000NsrF_H202A_P. An empty vector *pIL-SVCm* was also transformed into the NZ9000 strain and was used as a control (that excludes any possible effect of induction of the plasmid), and this strain was called NZ9000Cm. The expression of the *nsrfp* gene is regulated by the TCS NisR/NisK present in the NZ9000 strain genome.

### Expression of NsrFP and NsrF_H202A_P

The NZ9000NsrFP and NZ9000NsrF_H202A_P strains were grown in GM17 media supplemented with 5 μg/ml chloramphenicol. By the addition of nisin (final concentration of 1 ng/ml, which is equivalent to 0.3 nM), the expression was induced and the culture was further grown overnight. To analyze the expression, the cells were harvested at OD_600_ of 2.0 by centrifuging at 5000 × *g* for 30 min. The resulting pellet was resuspended with R-buffer [50 mM HEPES pH 8.0, 150 mM NaCl, 10% (w/v) glycerol] to an OD_600_ of 200. Then 1/3 (w/v) glass beads (0.3 mm) were added and cells were lysed. A cycle of 1 min disruption and 2 min cooling on ice was repeated five to six times. A low centrifugation step at 10,000 × *g* to collect the cytoplasmic part was performed. Followed by a high spin step (100,000 × *g*) to harvest the membranes. To collected cytoplasmic and membrane fractions SDS-loading dye [0.2 M Tris–HCl, pH 6.8, 10% (w/v) SDS, 40% (v/v) glycerol, 0.02% (w/v) bromophenol and β-mercaptoethanol] was added, samples were further used for SDS-PAGE and Western blot analysis (20 μl loaded). To detect NsrFP and NsrF_H202A_P a polyclonal antibody against the large extracellular loop of NsrP was used (Davids Biotechnologie, Regensburg, Germany).

### Cloning of the Nisin H and CCCCP Variant

The used variants (CCCCA, CCCAA, nisin_1-28_ and nisin_1-22_) were previously described in Alkhatib et al. (2014b). Nisin H (O’Connor et al., 2015) was created by introducing five point mutations into the *pNZ-SV-nisA* vector (Alkhatib et al., 2014b). For the I_1_F-L_6_M point mutations we used the following primers (forward: 5′-GTGCATCACCACGCTTTACAAGTATTTCGATGTGTACACCCGGTTG-3′; reverse: 5′-CAACCGGGTGTACACATCGAAATACTTGTAAAGCGTGGTGATGCAC-3′). The G_18_T-M_21_Y mutations were introduced with the primers (forward: 5′-GTAAAACAGGAGCTCTGATGACATGTAACTATAAAACAGCAACTTGTCATTG-3′; reverse: 5′-CAATGACAAGTTGCTGTTTTATAGTTACATGTCATCAGAGCTCCTGTTTTAC-3′) and the last mutation H_31_K with the primers (forward: 5′-CTTGTCATTGTAGTATTAAAGTAAGCAAATAAGCTTTC-3′; reverse: 5′-GAAAGCTTATTTGCTTACTTTAATACTACAATGACAAG-3′). The CCCCP variant, were the last cysteine was exchanged by a proline was created into the *pNZ-SV-nisA* vector with the primers (forward: 5′-CAGCAACTTGTCATCCAAGTATTCACGTAAG-3′; reverse: 5′-CTTACGTGAATACTTGGATGACAAGTTGCTG-3′).

The resulting plasmids were verified by sequencing and transformed into the *L. lactis* strain NZ9000 (already containing the *pIL3-BTC* vector; Rink et al., 2005) for expression by electroporation as described above.

### Expression, Purification of Prenisin Variants

Prenisin was purified as described in Alkhatib et al. (2014b). Activation of purified prenisin was done by overnight cleavage at 8°C with purified NisP (Abts et al., 2013). The efficiency of the reaction was monitored and the concentration of active nisin was determined by RP-HPLC as previously described (Abts et al., 2013). The activated nisin variants were then directly used for IC_50_ assays. Gallidermin is commercially available (Enzo Life Sciences).

### Purification of Nisin

Nisin was purified as described in Abts et al. (2011). The concentration of nisin was measured by using RP-HPLC as previously described (Abts et al., 2013).

### Determination the Activity of Nisin by Growth Inhibition (IC_50_)

Cells from the different expressing strains were grown overnight in GM17 supplemented with 5 μg/ml chloramphenicol in presence of 1 ng/ml nisin. The diluted cells (final OD_584_ was 0.1) were incubated with a serial dilution of nisin in a 96-well plate. The total volume in each well was 200 μl, consisting of 50 μl nisin and 150 μl GM17 containing the corresponding *L. lactis* strain. The highest concentration of nisin used was adapted to the corresponding maximum resistance displayed by each strain.

The plate was incubated at 30°C. After 5 h, the optical density was measured at 584 nm via 96-well plate reader BMG. The normalized optical density was plotted against the logarithm of the nisin concentration in order to calculate the IC_50_ of nisin and the data was evaluated using the following equation (Eq. 1):

$$\begin{array}{c} y={\mathrm{OD}}_{\min}+\frac{{\mathrm{OD}}_{\max}-{\mathrm{OD}}_{\min}}{1+10^{\left(\log({\mathrm{IC}}_{50})-x\right)\;\times p}} \end{array}$$

The OD_max_ value describes the normalized optical density value where no nisin was added, while the OD_min_ value corresponds to the normalized optical density of the cells grown in the highest nisin concentrations. The *y* represents the resulted normalized optical density value and *x* represents the logarithmic of the nisin concentration added. The IC_50_ value is the concentration of nisin where the growth of the *L. lactis* strain is inhibited by 50% (Abts et al., 2011).

### Calculation of the Fold of Resistance

We determined the IC_50_ value of nisin against the NZ9000Cm sensitive strain as well as the strain NZ9000NsrFP and NZ9000NsrF_H202A_P. By dividing these two values the fold of resistance is obtained. For example wild type nisin displayed an IC_50_ of 4.9 nM against NZ9000Cm and 82.2 nM against NZ9000NsrFP. Dividing these two values results in a fold of resistance of 16.7. We used this fold of resistance to obtain a quantitative, comparable value for the nisin variants.

### Dependency of Nisin Variants on Induced Expression of NsrFP

We verified the expression level of NsrFP in the corresponding strain NZ9000NsrFP by inducing expression with the different nisin variants. Here, we used half the IC_50_ value, which was determined for each nisin variant against the sensitive NZ9000Cm strain, to exclude an effect on the expression level of NsrFP. The initial OD_600_ of the NZ9000NsrFP strain was 0.1 and we induced each sample with the half IC_50_ value of the corresponding nisin variants. The strains were further grown for 5 h at 30°C. After harvesting the cells, SDS-PAGE samples were prepared as describe above. The expression of NsrFP was analyzed by Western blot using a polyclonal antibody directed against the extracellular loop.

### SYTOX Green Nucleic Acids Binding Assay

SYTOX green nucleic acids binding dye possesses a high binding affinity toward nucleic acids. It enters cells, which contain a pore in the plasma membrane and never crosses the intact membranes of living cells (Roth et al., 1997). The cells of NZ9000NsrFP were grown overnight in GM17 supplemented with 5 μg/ml chloramphenicol in presence of 1 ng/ml nisin. The next day, the overnight culture was diluted to an OD_600_ of 0.1 in fresh media supplemented with 5 μg/ml chloramphenicol. The cultures were grown until the OD_600_ reaches 0.5, the SYTOX green dye was added at a final concentration of 2.5 μM and incubated for 5 min according to the manual of the manufacturer (Invitrogen). The fluorescence signal, which was measured at an excitation and emission wavelength of 504 and 523 nm, respectively, was monitored. After a stable baseline is reached, nisin was added and the fluorescence was monitored over an additional time period.

### Nisin Transport Assay

To answer the question whether NsrFP is an importer or exporter we performed a well-known nisin transport assay (Stein et al., 2003, 2005).

We grew the cells of NZ9000NsrFP, NZ9000NsrF_H202A_P, and NZ9000Cm in GM17 supplemented with 5 μg/ml chloramphenicol in presence of 1 ng/ml nisin. We harvested the cells and washed them with 50 mM HEPES, pH 7, 500 mM NaCl, 10 % (v/v) glycerol. The cell density was adjusted to an OD_600_ of 10 in 1 ml of the corresponding strain and incubated with 8 μg nisin at 30°C for 30 min under gently shaking. After centrifugation at 10,000 × *g* for 10 min the supernatant was collected and applied to RP-HPLC for the quantification of nisin as described above.

## Results

### IC_50_ Determination of NZ9000NsrFP and NZ9000NsrF_H202A_P

**FIGURE 3:**
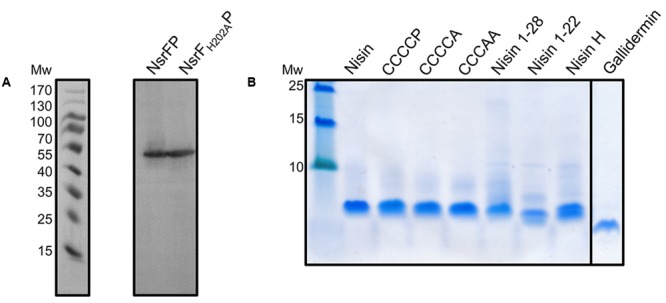
Expression of NsrFP and NsrF_H202A_P in *L. lactis* and purification of nisin variants. **(A)** The expression of NsrFP and NsrF_H202A_P was monitored by Western blot using a polyclonal antibody against the extracellular domain. As observed both proteins are expressed at a similar level in *L. lactis* NZ9000. **(B)** Nisin and its variants were purified and activated by using a standard procedure (see Materials and Methods). All substrates display a similar purity, judged by a 20% Tricine-SDS-PAGE.

We cloned NsrFP and NsrF_H202A_P in a *pIL-SVCm* shuttle vector and induced the expression with a sublethal amount of nisin (0.3 nM). To ensure, that there were no side effects from induction with nisin, we compared all experiments with a control strain. This strain was transformed with an empty plasmid and was treated exactly the same. We observed that the induction using 0.3 nM had no influence on the morphology or growth behavior of the *L. lactis* strains. This expression system has been used in the past for several proteins involved in nisin modification as well as immunity and resistance (Kuipers et al., 2004; Plat et al., 2011; AlKhatib et al., 2014a; Khosa et al., 2016a). Nisin was purified as previously described (**Figure 3**; Abts et al., 2011). To address the activity of nisin against the NZ9000Cm, NZ9000NsrFP, and NZ9000NsrF_H202A_P strains, growth experiments were performed using an increasing concentration of nisin. From these the IC_50_ values were determined, which reflects the growth inhibition of the corresponding strain by 50% using Eq. 1.

**FIGURE 4:**
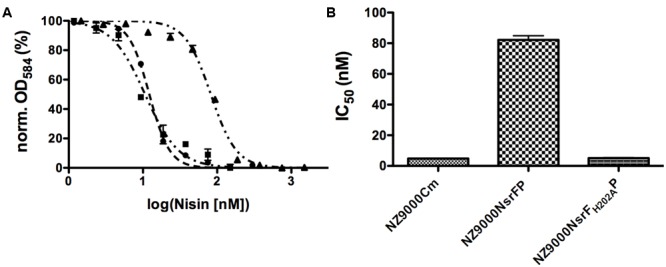
Activity of nisin against the NZ9000Cm, NZ9000NsrFP, and NZ9000NsrF_H202A_P strains. **(A)** The IC_50_ of nisin against the NZ9000Cm (●), NZ9000NsrFP (▲), and strain NZ9000NsrF_H202A_P (■) was determined. As observed the curve shifted to higher nisin concentration indicating that the NZ9000NsrFP strain is more resistant toward nisin. **(B)** The calculated IC_50_ values of nisin against the NZ9000Cm, NZ9000NsrFP, and the NZ9000NsrF_H202A_P strain are highlighted.

Nisin is highly active against the NZ9000Cm strain, as observed by the IC_50_ value of 4.9 ± 0.4 nM (**Figure 4** and **Table 1**). The NZ9000NsrFP strain exhibited a higher IC_50_ value of 82.2 ± 6.7 nM (**Figure 4** and **Table 1**). By dividing the two values a 16.7-fold of resistance was calculated (see Materials and Methods). This highlights that NsrFP expressed in *L. lactis* confers resistance against nisin. We cloned a variant of NsrFP termed NsrF_H202A_P, where the histidine at position 202 of NsrF is mutated to an alanine. By sequence alignments this histidine residue was identified as the catalytically important residue for ATP hydrolysis, generally termed as H-loop (Zaitseva et al., 2005). The corresponding NZ9000NsrF_H202A_P strain displayed a lower IC_50_ value of 5.1 ± 0.8 nM, which within experimental error represents the same value as obtained for the NZ9000Cm strain (**Figure 4** and **Table 1**). This suggests that NsrFP relies on ATP hydrolysis to confer resistance against nisin. Here, we have to note that the expression of NsrF_H202A_P led to a reduced final OD (0.8 compared to 1.1 for the wild type strain) in our growth experiments. The observed difference does not rise from different expression levels of NsrFP and the NsrF_H202A_P variant as shown by Western blot analysis using a polyclonal antibody directed against the large ECD of NsrP (Davids Biotechnology, Regensburg, Germany) (**Figure 3A**).

**Table 1:** IC_50_ values of nisin and its variants against the NZ9000Cm, NZ9000NsrFP, and NZ9000NsrF_H202A_P strains.

	NZ9000Cm	NZ9000NsrFP		NZ9000NsrF_H202A_P	
Nisin variant	IC_50_ (nM)	IC_50_ (nM)	Fold of resistance	IC_50_ (nM)	Fold of resistance
Wild type	4.9 ± 0.4	82.2 ± 6.7	16.7	5.1 ± 0.8	1.1
CCCCP	39.7 ± 1.5	238.4 ± 11.7	6.0	37.8 ± 3.9	0.9
CCCCA	64.4 ± 8.4	2023 ± 143	31.4	38.9 ± 5.9	0.6
CCCAA	278.6 ± 18.8	36346 ± 3632	130.5	154.6 ± 30.8	0.5
Nisin_1-28_	157.0 ± 8.7	5243 ± 1225	33.4	65.3 ± 11.4	0.4
Nisin_1-22_	309.9 ± 51.4	12220 ± 804	39.4	209.0 ± 39.9	0.7
Nisin H	7.0 ± 0.4	86.5 ± 3.7	12.3	7.5 ± 0.8	1.1
Gallidermin	67.1 ± 9.1	840 ± 87.0	12.5	59.7 ± 7.3	0.9

*Besides the IC_50_ values also the fold of resistance against the nisin variants mediated by NsrFP are shown. The fold of resistance is calculated by the division of the IC_50_ value obtained of the NZ9000NsrFP by the value for the NZ9000Cm strain. The values represent the average and standard deviation of at least four different experiments.*

### Pore Formation of Nisin in the NZ9000NsrFP Strain

**FIGURE 5:**
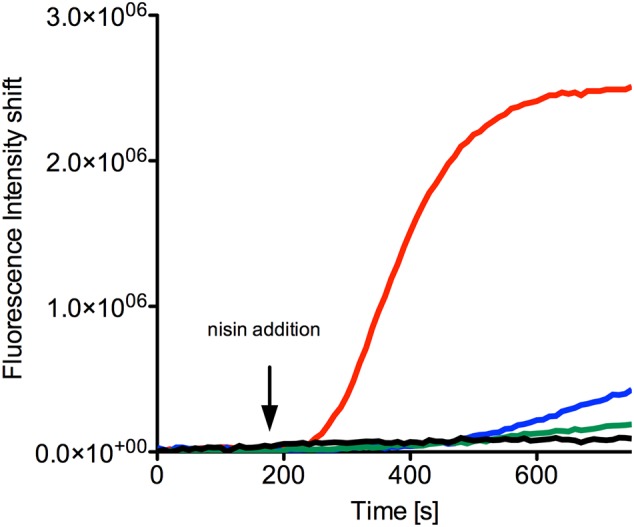
SYTOX green assay to visualize pore formation mediated by nisin. The NZ9000NsrFP strain was grown until OD_600_ of 0.5 and then incubated with the SYTOX dye. After a stable baseline was reached nisin (indicated with an arrow) was added at various concentrations: 40 nM (green line), 80 nM (blue line), and 160 nM (red line). As a control only buffer was added (black line). The fluorescence signal was monitored online using a fluorolog (Horiba III) and the rapid increase indicates pore formation. The curves are representatives of at least four biological replicates.

Nisin is able to form pores in the membrane of Gram-positive bacteria initiated by the initial binding to lipid II and subsequently reorientation of the C-terminal part of nisin into the membrane (Hasper et al., 2004). This leads to membrane leakage and rapid cell death. We monitored this pore formation using a SYTOX green nucleic acid dye (Roth et al., 1997). When pores are formed in the membrane the SYTOX dye enters the cells and binds to the DNA, resulting in an increased fluorescence signal. This is an almost instant effect, which can be monitored in real time. We monitored the pore forming action of nisin against the NZ9000NsrFP, using different nisin concentrations, which were based on the IC_50_ values of the corresponding strains determined above. As a control, we added only buffer without nisin, which resulted in no increase of the fluorescence signal as observed by the black line in **Figure 5**. This control indicates that no cells are spontaneously lysed under this experimental setup.

When 40 nM nisin (corresponding to half the IC_50_ value determined for the NZ9000NsrFP strain) was added to the NZ9000NsrFP strain, no increase of the fluorescence signal was observed (**Figure 5**, green line). This indicates that the NZ9000NsrFP strain can survive a nisin concentration of 40 nM. Only a small linear increase was visible after 400 s, which reflects to a less extent cell lysis after some time. A nisin concentration equivalent to the IC_50_ value (80 nM) resulted in a slightly stronger increase of the signal after a delay time (**Figure 5**, blue line). Finally, after adding a nisin concentration of two-times the IC_50_ value (e.g., 160 nM to the NZ9000NsrFP strain) a rapid increase of the fluorescence signal was observed and reaches a stable plateau already after a couple of seconds. This shows that NsrFP is not able to confer resistance above the determined IC_50_ concentration (**Figure 5**, red line).

### Nisin Transport Assay–Peptide Release Assay

**FIGURE 6:**
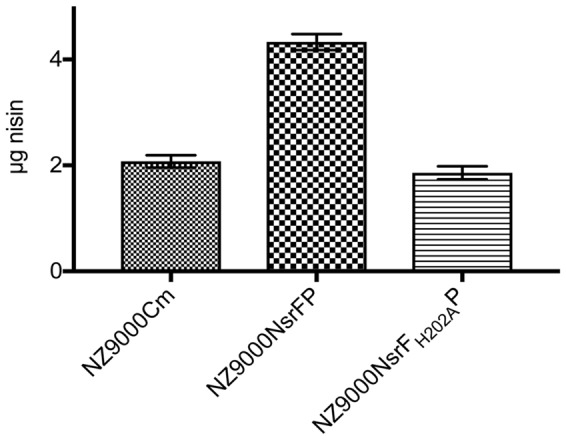
Nisin transport assay. The NZ9000Cm, NZ9000NsrFP, and NZ9000NsrF_H202A_P strain were incubated with 8 μg nisin. After incubation for 30 min the cells were spun down and the amount of nisin in the supernatant was determined using RP-HPLC. The NZ9000Cm and NZ9000NsrF_H202A_P strain showed similar nisin amounts. The NZ9000NsrFP strain revealed a ∼2-fold increased nisin amount, highlighting that NsrFP exports nisin from the *L lactis* membrane.

We performed a peptide release assay to verify the transport direction of NsrFP. Previously, the same assay was used to characterize NisFEG and SpaFEG, two exporting systems from lantibiotic producing strains (Stein et al., 2003, 2005). Here, we incubated the NsrFP expressing strain with 8 μg nisin for 30 min. After centrifugation of the cell, the supernatant was analyzed via RP-HPLC to determine the amount of nisin. From 8 μg nisin, 4.3 μg nisin was recovered from the supernatant (**Figure 6**). As a control, we used the NsrF_H202A_P and the sensitive NZ9000Cm strain. There only ∼2 μg nisin was recovered from the supernatant (**Figure 6**). This shows that NsrFP is able to export nisin from the cellular membrane. Our results are similar to the results found for the NisFEG and SpaFEG transporters leading to the same conclusion that NsrFP is exporting nisin from the cellular membrane as well.

### Substrate Specificity of NsrFP

In order to investigate the substrate specificity of NsrFP we used a set of nisin variants. Here, the nisin variants CCCCA, CCCAA, nisin_1-28_, and nisin_1-22_ were used (Khosa et al., 2016a). These variants are lacking the last or last two lanthionine rings or display deletions at the C-terminus of nisin, respectively. CCCCP is a variant, where the cysteine at position 28 (important for ring E formation) is exchanged to a proline (for a schematic view see **Figure 1**).

**FIGURE 7:**
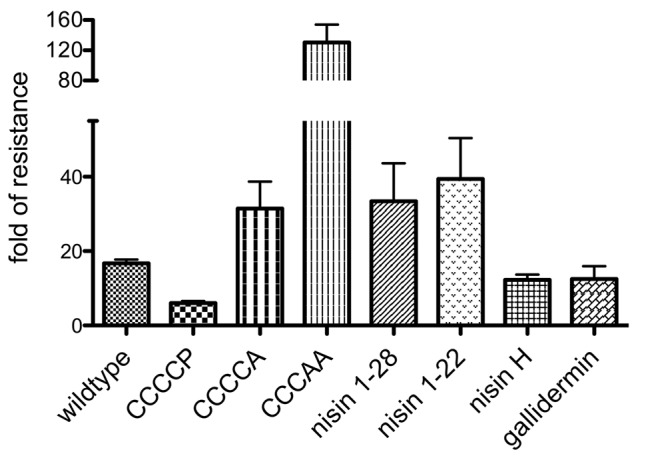
Substrate specificity of NsrFP. Graphical representation of the fold of resistance exhibited by NsrFP with nisin and different nisin variants (CCCCA, CCCAA, CCCCP, nisin_1-22_ and nisin_1-28_) as well as nisin H and gallidermin. The NZ9000Cm and NZ9000NsrFP strains were used to determine the activity of all tested lantibiotics. The error bars indicate the standard error of at least three independent experiments.

Expression and purification were performed as previously described (Alkhatib et al., 2014b), resulting in high purity (**Figure 3B**). The activities of these variants were determined against the nisin sensitive NZ9000Cm strain and the strains expressing NsrFP or NsrF_H202A_P, respectively (**Table 1**). By comparing these values the fold of resistance was obtained (**Figure 7**) as determined for the wild type nisin (see above and Materials and Methods).

For CCCCP, the fold of resistance was determined to be 6.0 (IC_50_ against NZ9000Cm was 39.7 ± 1.5 nM and against NZ9000NsrFP was 238.4 ± 11.7 nM). For CCCCA, the fold of resistance was determined to be 31.4 (IC_50_ against NZ9000Cm was 64.4 ± 8.4 nM and against NZ9000NsrFP 2023 ± 143 nM). The CCCAA variants displayed a 130.5-fold of resistance (IC_50_ against NZ9000Cm was 278.6 ± 18.8 nM and against NZ9000NsrFP 36346 ± 3632 nM). The two deletion mutants displayed a 33.4 (nisin_1-28_) and 39.4 (nisin_1-22_) fold of resistance, almost five times higher when compared to wild type. Here IC_50_ were determined to be 157 ± 8.7 nM against NZ9000Cm and 5243 ± 1225 nM against NZ9000NsrFP strain for nisin_1-28_ and 309.9 ± 51.4 nM against NZ9000Cm and 12,220 ± 804 nM against NZ9000NsrFP for nisin_1-22_, respectively.

These results revealed that NsrFP is able to be active as long as the N-terminal region of nisin is present, and since this part is highly conserved in several other lantibiotics, we hypothesized that the NsrFP transporter can besides nisin also recognize other lantibiotics. To test this, we used two other lantibiotics: nisin H (O’Connor et al., 2015) and gallidermin which is produced by *Staphylococcus gallinarum* Tü3928 (Kellner et al., 1988) (schematically shown in **Figure 1**). The latter contains a similar N-terminal part but has in comparison to nisin a structurally non-related C-terminus.

We determined the fold of resistance mediated by NsrFP for these lantibiotics (**Table 1** and **Figure 7**). Here, it was observed that nisin H as well as gallidermin are also recognized and NsrFP confers resistance to these lantibiotics. Our calculated fold of resistance is 12.3 for nisin H and 12.5 for gallidermin (**Table 1**). This strengthens the observation that the N-terminal region plays a predominant role in substrate recognition, since nisin H and gallidermin are recognized as well with similar efficiencies.

**FIGURE 8:**
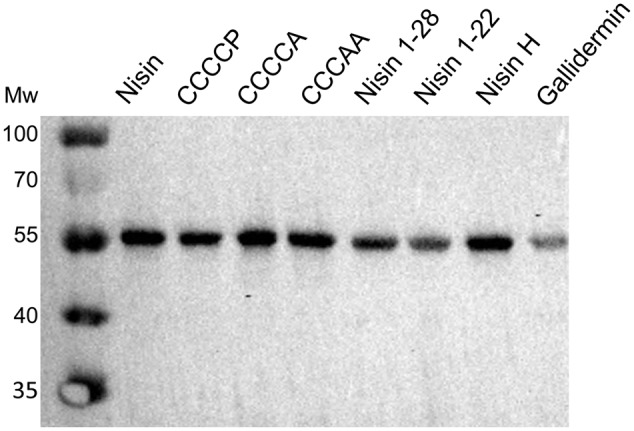
Dependency of nisin variants on induced expression of NsrFP. The expression of NsrFP was monitored by Western blot using a polyclonal antibody against the extracellular domain. As observed, the expression of NsrFP was similar in all cases, independent of the used nisin variant.

We were wondering whether the effect of a higher fold of resistance actually was a result of an increased expression of NsrFP in the membrane. Therefore, we incubated NZ9000NsrFP cells with the corresponding nisin variants (note: the concentration is 1/2 IC_50_ value of each nisin variant) and visualized the expression of NsrFP by Western blot. Here, we observed that the expression levels of NsrFP were similar for each strain and thereby cannot be the reason for the higher increased fold of resistance (**Figure 8**).

## Discussion

Lantibiotics possess antimicrobial activity against various bacteria including the well known MRSA, VISA, and VRE strains (Piper et al., 2009). However, various bacteria, especially human pathogens are actually inherently resistant against lantibiotics, which they do not produce themselves. Interestingly, this resistance is often arising from the expression of one or two membrane embedded proteins. Here, one belongs to the BceAB-type ABC transporter family and confer resistance against antimicrobial peptides including lantibiotics (Kallenberg et al., 2013; Kingston et al., 2014). Genetically the BceAB-type transporters are often located next to a TCS in the genome, which regulates the expression of the genes encoded (Dintner et al., 2011). It is thought that especially the ECD, which is a hallmark of BceAB-type ABC transporters is involved in lantibiotic sensing and transferring the signal to the corresponding histidine kinase (Staroń et al., 2011; Kallenberg et al., 2013). We focused on the nisin resistance operon from the *S. agalactiae* COH1, more specifically the BceAB-type ABC transporter NsrFP (Khosa et al., 2013). This transporter is localized on a gene operon together with the membrane associated protease NSR and the TCS NsrR and NsrK (Khosa et al., 2013, 2016b). We heterologously expressed the transporter in *L. lactis*, which lacks the NsrR/NsrK TCS and observed that NsrFP is able to confer resistance by itself. The fold of resistance, which we used as a measure of the activity, revealed that the *L. lactis* cells are able to deal with a 16-fold higher nisin concentration when compared to the same strain lacking NsrFP. The fold of resistance of an ATP hydrolysis deficient mutant of NsrFP is reduced to levels observed for the nisin sensitive NZ9000Cm strain. Like NisFEG (Stein et al., 2003) and SpaFEG (Stein et al., 2005), NsrFP acts as an lantibiotic exporter, which so far has not been conclusively shown for an lantibiotic resistance ABC transporter.

Intriguing is the observation that the N-terminal part of nisin appears to be important for NsrFP. By using C-terminal variants and deletions of nisin the fold of resistance increased in comparison to the wild type nisin. Only the variant CCCCP displayed a reduced fold of resistance. The recognition of the N-terminal region was further underlined by the observation that nisin H as well as gallidermin were also recognized as substrates. Here, especially the latter is containing a similar N-terminal region but differs structurally completely at the C-terminus (**Figure 1**).

Previously, the recognition of ring A and B was observed for the lantibiotic resistance ABC transporter CprABC from *Clostridium difficile*, which recognizes multiple lantibiotics: for example, nisin, gallidermin, subtilin, and mutacin 1140 (McBride and Sonenshein, 2011; Suárez et al., 2013).

Within the nisin resistance operon in *S. agalactiae* COH1 two proteins, namely the membrane associated protease NSR and NsrFP, are present (Khosa et al., 2013, 2016b). NSR is cleaving off the last six amino acids of nisin resulting in nisin_1-28_, which has a 32-fold lower activity. This product of NSR (nisin_1-28_), however, is still well recognized by NsrFP, as shown by an even increased fold of resistance. This suggests that both proteins are working together to obtain full resistance in *S. agalactiae*. The first line of defense would be NSR and the resulting processed product nisin_1-28_, is transported by NsrFP, once it reaches the membrane with high efficiency. This type of cooperativity would be similar to the natural immunity system observed in the nisin and subtilin (auto)immunity systems from *L. lactis* and *B. subtilis*, respectively. There, a cooperative mode of action of the immunity proteins LanI and LanFEG have been observed by which only full immunity was displayed when both protein are simultaneously expressed (Stein et al., 2003, 2005). Therefore, we suggest that NsrFP and NSR have a similar cooperative mode of action and only when both proteins are expressed simultaneously within the membrane of *S. agalactiae* COH1 full resistance is occurring.

## Author Contributions

SS conceived and directed this study. JR, ML, SL, and KE conducted the experiments. JZ-K established the mutants of nisin and created the figures. JR, ML, and SS wrote the manuscript with input of all authors. All authors read and approved the manuscript.

## Conflict of Interest Statement

The authors declare that the research was conducted in the absence of any commercial or financial relationships that could be construed as a potential conflict of interest.
